# Update on Inherited Pediatric Motor Neuron Diseases: Clinical Features and Outcome

**DOI:** 10.3390/genes15101346

**Published:** 2024-10-21

**Authors:** Antonio Trabacca, Camilla Ferrante, Maria Carmela Oliva, Isabella Fanizza, Ivana Gallo, Marta De Rinaldis

**Affiliations:** 1 Scientific Institute IRCCS. “E. Medea”, Scientific Direction, 23842 Bosisio Parini, Italy; 2 Associazione “La Nostra Famiglia”, IRCCS “E. Medea”, Scientific Hospital for Neurorehabilitation, Unit for Severe Disabilities in Developmental Age and Young Adults, Developmental Neurology and Neurorehabilitation, 72100 Brindisi, Italy; camilla.ferrante@lanostrafamiglia.it (C.F.); mariacarmela.oliva@lanostrafamiglia.it (M.C.O.); isabella.fanizza@lanostrafamiglia.it (I.F.); ivana.gallo@lanostrafamiglia.it (I.G.); marta.derinaldis@lanostrafamiglia.it (M.D.R.)

**Keywords:** motor neuron diseases, inheritance, genetic, children, whole-exome sequencing, whole-genome sequencing, spinal muscular atrophy, spinal bulbar muscular atrophy, hereditary spastic paraplegia, amyotrophic lateral sclerosis

## Abstract

Background: Inherited pediatric motor neuron diseases (MNDs) are a group of neurodegenerative disorders characterized by the degeneration of motor neurons in the brain and the spinal cord. These diseases can manifest as early as infancy and originate from inherited pathogenic mutations in known genes. Key clinical features of MNDs include muscle weakness, hypotonia, and atrophy due to the degeneration of lower motor neurons or spasticity, hypertonia, and hyperreflexia caused by upper motor neuron dysfunction. The course of the disease varies among individuals and is influenced by the specific subtype. Methods: We performed a non-systematic, narrative clinical review, employing a systematic methodology for the literature search and article selection to delineate the features of hereditary pediatric motor neuron diseases. Results: The growing availability of advanced molecular testing, such as whole-exome sequencing (WES) and whole-genome sequencing (WGS), has expanded the range of identified genetic factors. These advancements provide insights into the genetic complexity and underlying mechanisms of these disorders. As more MND-related genes are discovered, the accumulating genetic data will help prioritize promising candidate genes for future research. In some cases, targeted treatments based on specific genetic mechanisms have already emerged, underscoring the critical role of early and timely diagnosis in improving patient outcomes. Common MNDs include amyotrophic lateral sclerosis, spinal muscular atrophy, and bulbar spinal muscular atrophy. Conclusion: This narrative clinical review covers the clinical presentation, genetics, molecular features, and pathophysiology of inherited pediatric MNDs.

## 1. Introduction

**Figure 1:**
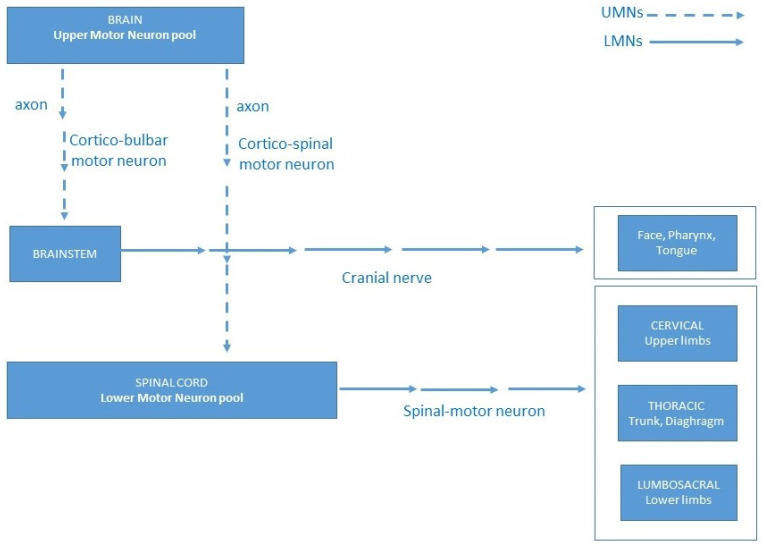
A schematic depiction of neuroanatomical structures illustrating the clinical link between descending corticospinal and corticobulbar motor neurons (upper motor neurons) and ascending spinal and bulbar motor neurons (lower motor neurons). The upper motor neurons (shown by dashed arrows) originate in the primary motor cortex and convey signals downward via their axons in the corticospinal tract to stimulate interneurons and lower motor neurons. Conversely, the lower motor neurons (depicted by continuous arrows) arise from either the anterior horn of the spinal cord or the cranial nerve nuclei in the brainstem, conveying signals from the upper motor neurons to the effectors.

Motor neuron disease (MND) is an umbrella term for a phenotypically heterogeneous group of progressive and, in some types, inevitably fatal disorders that affect primarily motor neurons (MNs). MNs are cells responsible for controlling the activity of skeletal muscles necessary for voluntary actions, such as walking, breathing, speaking, and swallowing. MNs are classified into two main types: upper MNs (UMNs) and lower MNs (LMNs) [1]. UMNs originate in the primary motor cortex and convey signals via their axons through the corticospinal tract to stimulate interneurons and lower motor neurons. LMNs originate in the anterior horn of the spinal cord or in the cranial nerve nuclei of the brainstem [2]. A subtype of these lower motor neurons, referred to as spine motor neurons (SpMNs), is situated in the ventral horn of the spinal cord and governs effector muscles in peripheral areas (Figure 1) [3].

This motor pathway organization helps classify motor neuron disorders into three categories: UMN disorders, LMN disorders, and mixed forms involving both [4].

The key clinical features of MND include muscle weakness, hypotonia, and atrophy, which are primarily due to the degeneration of LMNs, or spasticity, hypertonia, and hyperreflexia caused by UMN dysfunction. The course of MND varies among individuals and is influenced by the specific subtype of the disease. Initially, MNDs may involve degeneration in a specific group of motor neurons, leading to dysfunction in a localized muscle group. However, as the disease progresses, degeneration often spreads, affecting a broader range of muscles and leading to more widespread impairment [5].

These diseases can manifest as early as infancy, originating from inherited pathogenic mutations in known genes, and they often persist into adulthood, accounting for approximately 5% of cases [6]. They may be hereditary or acquired and are classified as rare diseases, also known as orphan diseases. These condition often present with severe, life-threatening symptoms in early childhood, posing significant challenges for patients and their families and contributing to the overall burden of disease. The overall prevalence rate, including adults and children, of certain MNDs, including amyotrophic lateral sclerosis (ALS), spinal muscular atrophy (SMA), spinal and bulbar muscular atrophy (SBMA), and hereditary spastic paraplegia (HSP), are approximately 4.5/100,000, 1–2/100,000, 1–2/100,000, and 1.8/100,000 inhabitants, respectively [7].

The growing availability of advanced molecular testing, such as whole exome sequencing (WES) and whole genome sequencing (WGS), has expanded the range of genetic factors identified in these conditions. WES and WGS have shown enhanced diagnostic efficacy in juvenile neurogenetic disorders [8]. Omics sciences are swiftly revolutionizing clinical research on these disorders, offering enhanced insights into the molecular processes involved and facilitating the categorization of individuals into diagnostic, prognostic, and therapeutic groupings. This has revealed broader and increasingly complex genetic variability, with significant overlap in clinical presentations [9,10,11].

With recent advancements in pharmaceutical treatments for various inherited disorders, it is crucial to understand these conditions, ensure timely diagnosis, and begin treatment without delay to achieve optimal long-term neurological outcomes [12,13].

This clinical review summarizes current knowledge on inherited pediatric NMDs with a focus on their clinical and epidemiological characteristics, diagnostic considerations, and genetic aspects (summarized in Table 1). It also addresses gene-specific management of causal genes and investigates the underlying processes of motor neuron degeneration where relevant. Prognostic factors and innovative therapies are also considered. For certain diseases, new and innovative therapies that can modify the prognosis, course, and clinical history are described, emphasizing the importance of a specific genetic diagnosis and timely treatment. 

## 2. Methods for Literature Search

The study was designed as a non-systematic, narrative clinical review utilizing a systematic approach for the literature search and article selection process. This methodology adheres to recognized guidelines for reporting narrative reviews [14].

The literature search was conducted using three major scientific databases, NCBI/PubMed, ScienceDirect, and Scopus, covering publications available up to June 2024. Search terms included a combination of broad and specific terms related to motor neuron disorders, such as “motor neuron disease”, “motor neuron disorders”, “amyotrophic lateral sclerosis”, “spinal muscular atrophy”, “spinal bulbar muscular atrophy”, “hereditary spastic paraplegia”, “SMA-like motor neuron disorders”, “SMA-plus”, “atypical SMA”, and “spinal muscular atrophy with respiratory distress”. Boolean operators were applied to effectively combine these terms, and the search strategy was tailored to the syntax requirements of each database.

Only articles written in English and reporting on the clinical features, outcomes, diagnosis, or treatment of motor neuron diseases in pediatric populations were included. Exclusion criteria comprised conference abstracts, editorials, news articles, opinion pieces, discussion papers, and studies focusing exclusively on adult patients. Non-peer-reviewed articles and papers lacking original data were also excluded from the review. To ensure comprehensive coverage, the search was supplemented by manually reviewing the reference lists of key articles. However, gray literature and unpublished studies were not included.

Rayyan, a web-based tool designed for systematic reviews, was used to manage the article selection process [15]. After the initial database search, articles were screened by title and abstract to exclude those that did not meet the inclusion criteria. Duplicates were automatically removed using Rayyan. Two independent reviewers conducted a full-text review of the selected studies to ensure they met the predefined inclusion criteria. In cases of disagreement, the software’s “blind” function allowed independent decisions before discussions to reach a consensus. A third reviewer was consulted to resolve any discrepancies. Only studies that met all inclusion criteria were included in the final analysis. The resulting literature was critically evaluated for relevance to the clinical features and outcomes of inherited pediatric motor neuron diseases.

In total, 105 articles were deemed eligible and included in the final analysis, focusing on the clinical features and outcomes of inherited pediatric motor neuron diseases.

## 3. Diagnostic and Management Challenges

The diagnosis of MNDs in children presents significant and complex challenges due to the wide spectrum of disorders, overlapping phenotypes, and diverse genetic factors.

Muscle atrophy or hypotrophy (amyotrophy) along with hypotonia, decreased reflexes, muscle weakness, and fasciculations, generally not accompanied by sensory deficits, are primary signs and symptoms of MNDs. The intensity of these symptoms may fluctuate based on the stage and advancement of the illness. When evaluating a pediatric patient for suspected motor neuron disease, the diagnostic process begins with a comprehensive review of the patient’s medical and family history, followed by a detailed neurological examination. Key aspects of the neurological assessment include evaluating the type and onset of weakness, amyotrophy, and the characteristics of progression (whether proximal or distal, bulbar or respiratory) [6]. Assessing deep tendon reflexes and muscle tone is also a critical component. Should the child’s clinical history and neurological assessment indicate a motor neuron disorder, a series of tests may be performed, including serum creatine kinase (CK) levels, thyroid function evaluations, cerebrospinal fluid (CSF) analysis for protein, inflammatory cells, microbial assessment, and metabolic screening and electrophysiological examination, such as electromyography (EMG) and nerve conduction velocity (NCV), to confirm the diagnosis. Serum CK levels may remain within the normal range or show a mild increase, usually not exceeding three times the upper limit of normal. EMG and NCV are essential for confirming a neurogenic origin and distinguishing motor neuron diseases from myopathies or disorders affecting the neuromuscular junction. They are capable of identifying both acute and chronic denervation [1]. While motor unit number estimation (MUNE) and motor unit number index (MUNIX) were once primarily used in research, they are now emerging as valuable biomarkers in spinal muscular atrophy and other motor neuron diseases [16,17,18].

A thorough evaluation of motor neuron diseases requires a multisystemic and multidisciplinary approach. This involves supplementary diagnostic tests, including cardiological, pulmonological, ophthalmological, and audiological assessments, as well as neurodevelopmental evaluations for children, alongside examinations of other general systems. These contribute to developing a comprehensive phenotypic profile. Neuroimaging of the brain and spinal cord further enhances and integrates the clinical assessment [19].

Second-level investigations should encompass genetic testing. Recent advances in DNA sequencing technology provide researchers unparalleled opportunities for genetic analysis. Next-generation sequencing (NGS), which supports whole-exome sequencing (WES) and whole-genome sequencing (WGS), has transformed the diagnosis of hereditary pediatric motor neuron disorders [20]. These innovations are reshaping clinical practice by becoming primary diagnostic tools, enabling more rapid diagnoses. Additional second-level assessments may involve muscle or nerve biopsies, muscle imaging, and metabolic testing [21,22].

## 4. Molecular Mechanisms and Pathophysiology

**Figure 2:**
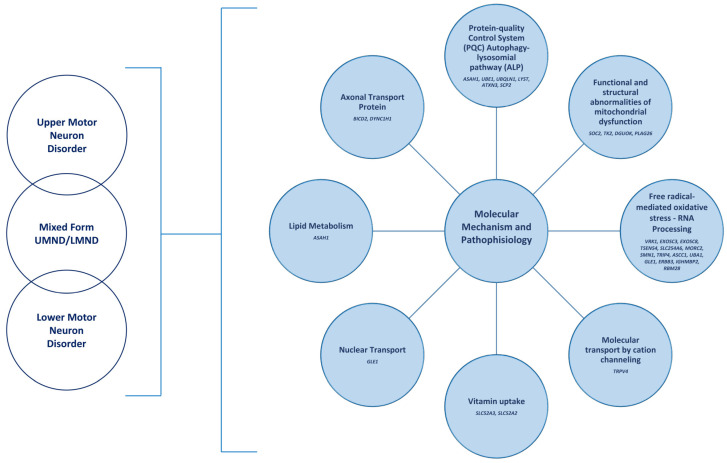
Molecular mechanisms and pathophysiology.

Understanding the molecular mechanisms and pathophysiology of motor neuron disorders (MNDs) is critical for managing these conditions and evaluating the implications of emerging disease-modifying therapies. Despite variations in symptoms, age of start, and development across motor neuron diseases (MNDs), the predominant pathogenic mechanism shared by most MNDs is the disruption of proteostasis and subsequent proteotoxicity [23]. This pathomechanism may be directly associated with mutations in genes that encode proteins integral to the protein quality control system (PQC), specifically the autophagy–lysosomal pathway (ALP), which is responsible for the elimination of cytosolic components, such as protein aggregates or damaged organelles (*ASAH1*, *UBE1*, *UBQLN1*, *LYST*, *ATXN3*, and *SCP2*) [24]. Autophagy is a crucial physiological mechanism that sustains proteostasis in neurons and other cell types commonly impacted in MNDs [25]. Its function in MNDs is highly intricate, with both inadequate and excessive activity contributing to the onset and/or advancement of various types of MNDs [5]. Furthermore, the reasons for this mechanism’s association with several clinical disorders remain ambiguous; other modifying elements may variably enhance or mitigate the harmful or beneficial effects of autophagy in each neuronal type [9]. Other processes involved in the pathophysiology of motor neuron diseases include structural and functional abnormalities of mitochondria (*SOC2*, *TK2*, *DGUOK*, and *PLAG26*), free-radical-mediated oxidative stress, RNA processing (*VRK1*, *EXOSC3*, *EXOSC8*, *TSEN54*, *SLC254A6*, *MORC2*, *SMN1*, *TRIP4*, *ASCC1*, *UBA1*, *GLE1*, *ERBB3*, *IGHMBP2*, and *RBM28*), cation-channel-mediated molecular transport (*TRPV4*), vitamin uptake (*SLC52A3* and *SLC52A2*), nuclear transport (*GLE1*), lipid metabolism (*ASAH1*), and axonal transport proteins (*BICD2* and *DYNC1H1*) [19,26,27]. The intersections between protein aggregation, stress granules, and autophagy are critical areas of study in understanding MNDs’ pathogenesis. Consequently, identifying an appropriate target for medications that might modulate autophagic pathways remains a challenge. Emerging evidence suggests that strategies designed to enhance pre-activated autophagy will yield more customized therapies to mitigate the neurotoxicity of aggregation-prone proteins in various motor neuron diseases associated with impaired responses to proteotoxic stress (Figure 2) [28].

## 5. MNDs with Prominent Lower Motor Neuron Involvement

### 5.1. Spinal Muscular Atrophy (SMA)

Inherited motor neuron disease in childhood is most frequently linked to mutations in the *SMN1* gene located on chromosome 5q13, resulting in spinal muscular atrophy (SMA). SMA encompasses a group of genetic disorders marked by progressive muscle weakness and atrophy caused by the degeneration of spinal motor neurons and, in more severe cases, the degeneration of lower bulbar motor neurons as well.

Spinal muscular atrophy (SMA) is a rare genetic disorder inherited in an autosomal recessive manner primarily affecting the spinal motor neurons [29]. It has an estimated prevalence of 2.12 per 100,000 individuals and is caused by the degeneration of alpha motor neurons in the spinal cord [30]. This leads to gradual muscle weakness and atrophy, particularly in the skeletal muscles. SMA is traditionally categorized into five phenotypes (types 0 through 4) differentiated by severity, motor milestones reached, and age of onset. Type 0 is the most severe, presenting before birth, while type 4 occurs in adulthood. The most common forms of SMA are types 1, 2, and 3, primarily affecting children [31].

SMA is generally caused by a homozygous mutation, deletion, or rearrangement in the survival motor neuron 1 (*SMN1*) gene on chromosome 5q13.3. These mutations cause deficiency of the ubiquitous SMN protein. In no other species save humans is the *SMN* gene duplicated to give rise to *SMN1* (telomeric form) and *SMN2* (centromeric form); *SMN2* differs from *SMN1* by a few nucleotides, the most crucial of which is a C to T transition in exon 7 that causes the skipping of this exon in > 90% of SMN2 transcripts. Consequently, *SMN2* mainly produces a truncated and unstable protein without exon 7, much of which is rapidly degraded. It should be noted, however, that SMN2 expression accounts for only 10% of the full-length fully functional SMN protein and thus only partially compensates for the loss of *SMN1*. *SMN2* is the key positive modulator of the SMA phenotype because the number of *SMN2* copies inversely correlates with the severity of the phenotype [32]. So, although most patients with SMA have deletions or mutations involving the *SMN1* gene, there is a range of phenotypic severity; this permits division into five clinical subtypes. The subtypes represent a phenotypic continuum extending from the very severe, with onset in utero, to the very mild, with onset during adulthood [31].

### 5.2. Type 0 SMA (Congenital SMA)

Patients with Type 0 SMA exhibit symptoms before birth, affecting the fetus during gestation. Decreased fetal movements are commonly observed prenatally. At birth, infants present with severe muscle weakness and typically experience respiratory failure. Death often occurs at birth or within the first month of life [33].

### 5.3. Type I SMA (Severe SMA or Werdnig–Hoffmann Disease)

Type I SMA symptoms manifest between birth and 6 months of age and are further subdivided into three categories: Type IA, where onset occurs in utero with symptoms present at birth; Type IB, where symptoms begin before 3 months of age; and Type IC, with onset between 3 and 6 months of age. Infants with Type I SMA experience progressive proximal muscle weakness, which is more pronounced in the legs than in the arms. They exhibit poor head control and hypotonia, leading to a “frog-leg” posture when lying down and a tendency to “slip through” when held vertically. Areflexia is also present. These infants are never able to sit or roll over independently and are referred to as “nonsitters”. Weakness of the intercostal muscles, with relative sparing of the diaphragm, leads to a bell-shaped chest and paradoxical or “belly” breathing patterns. Tongue fasciculations are characteristic, and difficulty swallowing may develop, contributing to failure to thrive and an increased risk of aspiration. Although other cranial nerves are less affected, facial weakness may occur in later stages. Without intervention, respiratory failure typically occurs by age 2 or earlier, though assisted ventilation has improved survival rates in recent years [34].

### 5.4. Type II SMA (Intermediate SMA or Dubowitz Disease)

Symptoms of Type II SMA typically present between 6 and 18 months of age. Patients experience progressive proximal muscle weakness, affecting the legs more than the arms, along with hypotonia and areflexia. Progressive scoliosis develops due to muscle weakness, contributing to significant restrictive lung disease. Joint contractures are common, and ankylosis of the mandible may occur. Patients often exhibit tremors or polyminimyoclonus in the hands. Cognitive development is normal, and verbal intelligence may be above average [35,36].

### 5.5. Type III SMA (Mild SMA or Kugelberg–Welander Disease)

Patients with Type III SMA are able to stand and walk independently at some point and are referred to as “walkers”. Symptom onset occurs after 18 months of age and is further subdivided into two categories: Type IIIA, with onset between 18 months and 3 years, and Type IIIB, with onset after 3 years. These patients experience progressive proximal muscle weakness, primarily affecting the legs more than the arms, and may eventually require wheelchair assistance, particularly in the Type IIIA group. They generally do not develop significant respiratory muscle weakness or severe scoliosis, though loss of ambulation increases the risk of these complications. Tremors or polyminimyoclonus of the hands may be present. Life expectancy is not significantly different from that of the normal population [37].

### 5.6. Type IV SMA (Adult Form)

Individuals diagnosed with SMA type 4, also known as adult-onset SMA, represent the milder end of the SMA range. Symptoms often manifest post-21 years of age; however, they may occasionally emerge earlier in adolescence. This variant accounts for fewer than 5% of all SMA cases and is deemed the least severe. Individuals with SMA type IV maintain ambulation and exhibit similarities to individuals with SMA type III. The illness advances gradually, with just a minor fraction losing the capacity for independent ambulation within 20 years after symptoms start. Fasciculations occur in around 75% of patients, sometimes accompanied by muscular cramps. Bulbar involvement and scoliosis are rare, and the intercostal muscles are often spared [38].

**Figure 3:**
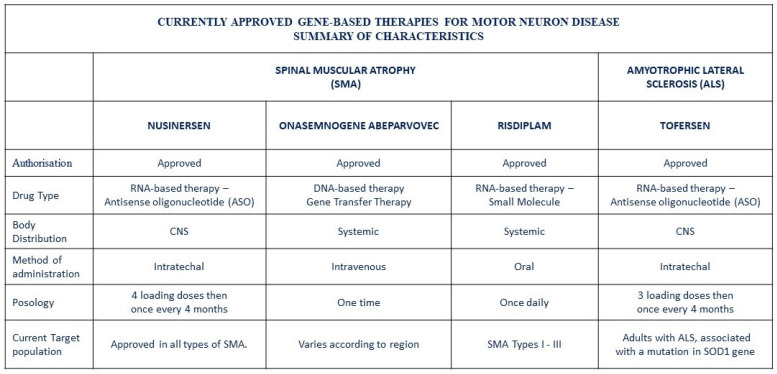
Currently approved gene-based therapies for motor neuron disease.

Three recently authorized gene-based therapies—nusinersen, onasemnogene abeparvovec, and risdiplam—have dramatically improved outcomes for patients with spinal muscular atrophy (SMA) (Figure 3) [39,40].

## 6. Summary of Characteristics

Nusinersen was the first SMA orphan drug approved by the U.S. Food and Drug Administration (FDA) in 2016 and by the European Medicines Agency (EMA) in 2017 for both children and adults. It is an RNA-based therapy classified as an antisense oligonucleotide (ASO). Nusinersen works by modifying the splicing of *SMN2* pre-mRNA, leading to increased production of full-length survival motor neuron (SMN) protein and demonstrating clinical efficacy. Specifically, it targets a splicing silencer site known as ISS-N1 within intron 7 of the *SMN2* gene. By preventing the binding of splicing repressors hnRNPA1 and hnRNPA2 to ISS-N1, nusinersen facilitates the inclusion of exon 7 in the final mRNA transcript, resulting in the synthesis of functional SMN protein. Due to its inability to cross the blood–brain barrier, nusinersen is administered via intrathecal injection. This treatment has significantly increased SMN protein levels, improved disease phenotypes, and extended median lifespans [41].

Onasemnogene abeparvovec, approved in 2020, is the first—and currently the only—gene replacement therapy for SMA. It is indicated for patients under two years old with bi-allelic mutations in the *SMN1* gene and three or fewer copies of the *SMN2* gene, or those with infantile-onset SMA. This DNA-based therapy replaces the defective *SMN1* gene using an adeno-associated viral vector (AAV9) capable of crossing the blood–brain barrier. A key advantage of this approach is that a single intravenous injection results in systemic expression of the SMN protein. However, potential drawbacks include a lack of long-term efficacy and safety data [42].

Risdiplam, an RNA-based therapy like nusinersen, modifies *SMN2* pre-mRNA splicing to increase SMN protein production. It is a small molecule that crosses the blood–brain barrier and is administered orally once daily, achieving bioavailability in both central and peripheral tissues. Risdiplam was approved by the FDA in 2020 and by the EMA in 2021 as the first oral drug for SMA treatment. The success of nusinersen spurred research into less invasive therapies, leading to the development of risdiplam, which promotes the inclusion of exon 7, similarly to nusinersen [43].

Advancements in therapeutic options have profoundly transformed the natural progression of spinal muscular atrophy (SMA), leading to new phenotypes characterized by reshaped symptom progression and increased survival rates. Patients are now reaching unprecedented motor milestones—such as sitting, standing, and walking with support—and are experiencing improved linguistic and communicative abilities. This is particularly remarkable for SMA type I patients, who previously often required tracheostomies. Rapid progress in clinical research, trials, and real-world evidence has accelerated our understanding of SMA’s natural history, prompting a revision of its phenotypic classifications. Currently, SMA is viewed as a continuum that emphasizes a patient’s functional status and response to therapy. Thanks to gene-based therapies, a patient diagnosed with SMA type I can now achieve functional capabilities similar to those of SMA types II, III, or IV. Similarly, a patient with SMA type II can attain abilities typically associated with SMA types III or IV [44].

## 7. MNDs with Prominent Lower Motor Neuron Involvement with Additional Features (Sma-like or Sma-Plus or Atypical Sma)

While mutations in the *SMN1* gene on chromosome 5q13 are the most common cause of inherited motor neuron disease in children, a small subset of patients (around 4%) do not exhibit *SMN1* mutations or deletions. Many of the genes implicated in these cases are involved in essential cellular processes, such as RNA processing, apoptosis, and the stress response, highlighting the vulnerability of motor neurons to these disruptions. While pediatric motor neuron diseases typically manifest as anterior horn cell disorders, some cases—classified as “SMA plus” or atypical SMA phenotypes—may present with additional symptoms, including epilepsy, encephalopathy, spasticity, or disturbances in gastrointestinal, respiratory, and rheumatologic systems. These syndromes, known as SMA plus or atypical SMA, are characterized by lower motor neuron dysfunction alongside other clinical features [45]

### 7.1. Spinal Muscular Atrophy with Respiratory Distress (SMARD)—Diaphragmatic SMA

Spinal Muscular Atrophy with Respiratory Distress (SMARD), also referred to as DSMA1 (Distal Spinal Muscular Atrophy with diaphragmatic paralysis), is a rare genetic disorder affecting infants [46]. Key early signs of SMARD include intrauterine growth restriction, a weak cry, difficulty sucking, and congenital foot deformities. These symptoms are often followed by respiratory failure caused by diaphragmatic paralysis and progressive muscle weakness. Typically, weakness and contractures first appear in the distal extremities. Unlike typical SMA, SMARD may also involve sensory and autonomic dysfunctions, such as excessive sweating, urinary retention, constipation, cardiac arrhythmias, and seizures. Two types of SMARD were identified.

SMARD1 is a rare autosomal recessive disorder caused by homozygous or compound heterozygous mutations in the IGHMBP2 gene on chromosome 11q13.3, which encodes immunoglobulin μ-binding protein 2 [47]. The exact function of this protein remains unclear, and it is unknown why mutations in this ubiquitously expressed protein selectively affect motor neurons. Variations in IGHMBP2 protein levels may explain differences in clinical presentations and outcomes [48].

SMARD2 is a phenotype observed in a single patient characterized by early diaphragmatic weakness, distal muscle weakness, and contractures. It is caused by an X-linked recessive mutation in the LAS1L gene on chromosome Xq12, which plays a role in ribosomal biogenesis [49].

### 7.2. SMA with Arthrogryposis

Infantile spinal muscular atrophy (SMA) with arthrogryposis presents clinical features indicative of prenatal onset of muscle weakness, which is often accompanied by abnormalities in bone and cardiac development. Common characteristics include a history of reduced fetal movements, polyhydramnios, breech presentation, pulmonary hypoplasia with diaphragmatic eventration, and early death. At birth, infants typically display arthrogryposis, osteopenia, multiple fractures, and congenital heart defects. Severe hypotonia, muscle weakness, areflexia, and tongue fasciculations are also observed. Muscle weakness leads to respiratory difficulties, kyphosis, scoliosis, mild micrognathia, and diminished facial expression. Most affected children do not survive beyond a few months due to respiratory failure unless they receive intensive respiratory and medical intervention [50]. To date, no disease-modifying treatments have been identified for congenital SMA with associated arthrogryposis or fractures. Recessive mutations in four distinct genes have been associated with congenital or infantile spinal muscular atrophy (SMA) accompanied by arthrogryposis or fractures. These genes include the following:

The survival motor neuron 1 (*SMN1*) gene;
The thyroid hormone receptor interactor 4 (*TRIP4*) gene;
The activating signal cointegrator 1 complex subunit 1 (*ASCC1*) gene;
The ubiquitin-like modifier-activating enzyme 1 (*UBA1*) gene.

These conditions are extremely rare, following either autosomal recessive or X-linked recessive inheritance patterns, with the available literature limited primarily to case reports.

### 7.3. X-Linked SMA and Arthrogryposis (XL-SMA, SMAX2)

X-linked spinal muscular atrophy with arthrogryposis (XL-SMA) is a severe genetic disorder primarily affecting boys due to mutations in the *UBE1* gene on chromosome Xp11.3. This condition is characterized by early-onset joint contractures (arthrogryposis) particularly impacting the proximal joints and fingers, alongside inwardly rotated arms and equinovarus foot deformities. Infants with XL-SMA often present with congenital hypotonia, areflexia, and progressive degeneration of lower motor neurons in the spinal cord and the brain stem. While intellect remains normal, respiratory failure due to chest muscle involvement typically leads to early mortality, with most affected children not surviving beyond age two. The disease follows an X-linked inheritance pattern, with a male predominance, and it can also involve more complex pathology in some cases [51,52].

### 7.4. Lethal Arthrogryposis with Anterior Horn Cell Disease (LAAHD) or Congenital Arthrogryposis with Anterior Horn Cell Disease; CAAHD

Lethal Arthrogryposis with Anterior Horn Cell Disease (LAAHD) is one of the most severe types of motor neuron diseases. This rare autosomal recessive disorder is caused by mutations in the *GLE1* gene, located on chromosome 9q34.11, which plays a crucial role in regulating RNA export. The *GLE1* gene encodes a protein responsible for transporting messenger RNA (mRNA) from the nucleus to the cytoplasm, a process critical for gene expression. This regulation affects various stages, including mRNA export, initiation of translation, and translation termination. Clinically, LAAHD is characterized by fetal immobility, multiple joint contractures (especially in distal joints, often with inward spiral deformities), low-set ears, a small or underdeveloped jaw (hypoplastic jaw), and a short neck. Some cases may also present with underdeveloped lungs (lung hypoplasia) and mild hydrops. The condition typically results in fetal death either in utero or shortly after birth. Pathologically, LAAHD is defined by degeneration and loss of anterior horn motor neurons, while the cerebrum and cerebellum remain unaffected [53].

### 7.5. Lethal Congenital Contracture Syndrome 1 (LCCS1) or Multiple Contracture Syndrome, Finnish Type

Lethal Congenital Contracture Syndrome 1 (LCCS1) is a severe autosomal recessive disorder caused by mutations in the *GLE1* gene, located on chromosome 9q34.11. This condition is marked by complete fetal immobility, severe hydrops, and significant intrauterine growth restriction, with most cases resulting in fetal death before 32 weeks of gestation [54].

### 7.6. Lethal Congenital Contracture Syndrome 2 (LCCS2) or Multiple Contracture Syndrome, Israeli Bedouin Type A

Lethal Congenital Contracture Syndrome 2 (LCCS2) is caused by mutations in the *ERBB3* gene on chromosome 12q13.2. This autosomal recessive syndrome is usually fatal shortly after birth and is characterized by cranial and ocular abnormalities, an enlarged bladder with hydronephrosis, and cystic kidney changes [55].

### 7.7. Lethal Congenital Contracture Syndrome 3 (LCCS3) or Multiple Contracture Syndrome, Israeli Bedouin Type B

Lethal Congenital Contracture Syndrome 3 (LCCS3), described by Narkis et al., resembles LCCS2 but lacks neurogenic bladder involvement. The gene responsible for this disease is located on chromosome 19p13.3 and causes a mutation in the *PIP5K1C* gene, which encodes phosphatidylinositol-4-phosphate 5-kinase type I gamma (PIPKIγ). This enzyme phosphorylates phosphatidylinositol 4-phosphate to produce phosphatidylinositol-4,5-bisphosphate (PIP2). As a result, a defect in the phosphatidylinositol pathway reduces the synthesis of PIP2, a key molecule in the endocytosis of synaptic vesicle proteins, ultimately leading to lethal congenital arthrogryposis [56].

### 7.8. Lethal Congenital Contractural Syndrome 4 (LCCS4)

Lethal Congenital Contractural Syndrome (LCCS4) is an autosomal recessive variant described by Markus et al. caused by a mutation in the *MYBPC1* gene, located on chromosome 12q23.2. The *MYBPC1* gene encodes for myosin-binding protein C, a myosin-associated skeletal protein found in the cross-bridge bearing zone (C region) of A bands in striated muscle. LCCS4 is a severe form of neuromuscular arthrogryposis characterized by contractures leading to various degrees of flexion or extension limitations evident at birth [57].

### 7.9. SMA Plus Syndromes Linked to Mitochondrial Disorders

SMA Plus syndromes associated with mitochondrial disorders are characterized by severe symptoms, including hypotonia, muscle weakness, and respiratory failure. These conditions may also involve infantile hypertrophic cardiomyopathy, liver failure, encephalopathy, and seizures. Mutations in several genes are commonly implicated, including the following.

The *SCO2* gene on chromosome 22q13 causes infantile-onset cardioencephalomyopathy and hypertrophic cardiomyopathy [58,59].

The *DGUOK* gene, associated with hepatocerebral syndrome (MTDPS3—mitochondrial DNA depletion syndrome 3), is characterized by liver failure, cerebral atrophy, and death by 12 months [60].

The *TK2* gene, linked to mitochondrial DNA depletion syndrome 2 (MTDPS2), usually presents as myopathy but can vary widely in clinical presentation [61].

### 7.10. Brown–Vialetto–Van Laere (BVVL) Syndrome

The Brown–Vialetto–Van Laere syndrome (BVVL) is a rare neurological disorder first described by Brown in 1894 and subsequently by Vialetto and Van Laere. Its prevalence is very low, with only 58 cases reported by 2008. Patients typically present with sensorineural deafness, bulbar palsy, and respiratory compromise, with the age of onset ranging from infancy to adulthood. Fazio–Londe syndrome shares the same clinical presentation but lacks the hearing loss; it is considered to be part of the same disease entity as BVVL [62]. Brown–Vialetto–Van Laere syndrome (BVVL) is a progressive autosomal recessive disorder that affects pontobulbar motor neurons, leading to symptoms like facial weakness, hearing loss, respiratory failure, and early death. It is caused by a homozygous or compound heterozygous mutation in the *C20ORF54* (*SLC52A3*) gene on chromosome 20p13, which encodes riboflavin transporters. High-dose riboflavin supplementation may offer clinical benefits [62,63].

As part of the genetic variability of Brown–Vialetto–Van Laere syndrome, other forms, such as Brown–Vialetto–Van Laere syndrome 2 (BVVLS2) and a variant linked to the *UBQLN1* gene, should also be considered. BVVLS2 is caused by a mutation in the *SLC52A2* gene on chromosome 8q24. It is an autosomal recessive neurological disorder characterized by early-onset sensorineural deafness, bulbar dysfunction, and severe muscle weakness and wasting in both upper and lower limbs, as well as the axial muscles, leading to respiratory insufficiency. Some affected individuals may lose the ability to walk independently. Because the condition is related to impaired riboflavin metabolism, high-dose riboflavin therapy may benefit some patients [64]. In 2012, Gonzalez-Perez and colleagues identified a heterozygous c.162G-T transversion in exon 1 of the *UBQLN1* gene in an Italian woman with motor neuron disease consistent with Brown–Vialetto–Van Laere syndrome [65].

### 7.11. Fazio-Londe Syndrome

Fazio–Londe syndrome is a condition similar to BVVL that presents with the same symptoms, except for hearing loss, and it is related to mutations in the *SLC52A3* gene located on chromosome 20p13, which encodes the intestinal riboflavin transporter (hRFT2). Additionally, some children with Fazio–Londe syndrome have shown improved outcomes following riboflavin therapy [66,67].

### 7.12. Pontocerebellar Hypoplasia with Spinal Muscular Atrophy (PCH1)

Pontocerebellar Hypoplasia with Spinal Muscular Atrophy (PCH1) is a neurodegenerative condition that belongs to a group of disorders characterized by spinocerebellar degeneration, cerebellar malformation, and significant brainstem hypoplasia. PCH type 1 is associated with anterior horn cell disease, which resembles infantile spinal muscular atrophy. Common symptoms of the condition include severe hypotonia, areflexia, muscle weakness, central visual impairment, dysphagia, respiratory insufficiency, and acquired microcephaly, often leading to early infant mortality. In some cases, patients may also experience ataxia and nystagmus [68]. The condition follows an autosomal recessive inheritance pattern, with mutations identified in specific genes. PCH type 1A is caused by mutations in the *VRK1* (vaccinia-related kinase 1) gene [69] located on chromosome 14q32.2, PCH type 1B is associated with mutations in the *EXOCS3* gene located on chromosome 9p13.2 [70], PCH type 1C is linked to mutations in the EXOCS8 gene located on chromosome 13q13.3 [71], and PCH type 1D caused by homozygous or compound heterozygous mutation in the *EXOSC9* gene on chromosome 4q27. In 2016, two additional genes related to SMAPCH1 were discovered. Mutations in the *SLC25A46* gene on chromosome 5q22.1, which encodes a mitochondrial protein, were found in families with pontocerebellar hypoplasia, optic atrophy, and sensorimotor neuropathy [72]. Additionally, a de novo *MORC2* mutation was identified in a single patient [73].

### 7.13. Spinal Muscular Atrophy with Progressive Myoclonic Epilepsy (SMAPME)

Spinal Muscular Atrophy with Progressive Myoclonic Epilepsy (SMAPME) is another rare condition that features childhood-onset progressive muscle weakness, hypotonia, areflexia, and myoclonic seizures. The EEG findings can vary, showing either a normal pattern or a slow background with epileptic activity, which may be photosensitive. This epileptic activity may correspond to clinical symptoms, such as limb jerks, head nodding, or negative myoclonus, with subcortical myoclonic epileptiform activity that is sensitive to hyperventilation. This form, transmitted in an autosomal recessive way, is caused by mutations in the *ASAH1* gene on chromosome 8p22, which encodes acid ceramidase, a lysosomal enzyme. Additional symptoms include mild facial weakness, tongue fasciculations, hearing loss, tremors, and recurrent respiratory infections. Life expectancy is typically limited to the first two decades due to respiratory complications. Currently, no disease-modifying treatments exist for SMAPME. While antiepileptic drugs can be used to manage myoclonic epilepsy, it often remains resistant to standard therapies [74].

### 7.14. Spinal Muscular Atrophy with Lower Extremity Predominance (SMA-LED)

Spinal Muscular Atrophy with Lower Extremity Predominance (SMA-LED) is characterized by a gradual progression of muscle weakness and atrophy primarily affecting the lower limbs, along with absent reflexes. Early signs include delayed walking, a waddling gait, and foot deformities, such as calcaneovalgus and pes planus. The condition typically presents at or before the age of five, with greater weakness in proximal muscles compared to distal muscles, particularly in the lower limbs. This muscle weakness and atrophy occur with normal sensation but lead to significant mobility impairments.

Common clinical features include delayed motor milestones, difficulty walking, a waddling gait, and absent distal reflexes. Additional complications may include congenital arthrogryposis, fasciculations, hip dysplasia, foot deformities like calcaneovalgus, pes planus, pes valgus, contractures, or hyperlordosis. The disorder is either non-progressive or only mildly progressive, and the degree of muscle wasting does not always correlate with the severity of muscle weakness. While early descriptions emphasized anterior horn cell disease, the spectrum of the condition has expanded to include other features, such as spasticity and cognitive impairment, suggesting a broader involvement of motor neurons. There are two types of SMA-LED, each caused by mutations in a different gene. SMA-LED1 is caused by mutations in the *DYNC1H1* gene on chromosome 14q32.31, while SMA-LED2 results from mutations in the *BICD2* gene on chromosome 9q22.31. Both genes are involved in axonal transport. SMA-LED1 and SMA-LED2 exhibit autosomal dominant inheritance [75,76].

### 7.15. Scapuloperoneal Spinal Muscular Atrophy (SPSMA)

Scapuloperoneal SMA (SPSMA) presents as a variable clinical disorder characterized by muscle absence, progressive muscle weakness, and atrophy, primarily affecting the scapula and lower limbs, and causing laryngeal palsy with vocal cord weakness, leading to hoarseness and respiratory stridor. Additional issues include torticollis, hip dysplasia, and delayed motor development, although intelligence remains normal. Symptoms, such as rounded shoulders, displaced scapulae, leg length discrepancy, hyperlordosis, and a wide-based gait, are also observed. By early adulthood, weakness and atrophy in the distal muscles become more pronounced. The condition progresses slowly, with muscle biopsies revealing neurogenic group fiber atrophy. SPSMA is an autosomal dominant condition caused by mutations in the *TRPV4* gene on chromosome 12q24.11, which encodes a calcium-permeable channel expressed in motor neurons, that are responsible for this subtype [77].

## 8. MNDs with Prominent Upper Motor Neuron Involvement

### Hereditary Spastic Paraplegia (HSP)

Hereditary spastic paraplegias (HSPs) are a group of rare inherited neurological diseases characterized by extreme heterogeneity in clinical manifestations and in genetic backgrounds characterized mainly by progressive lower extremity spasticity and weakness. An increasing number of HSP subtypes are being identified, each associated with distinct causal genes, unique phenotypes, and specific genotype–phenotype correlations. These subtypes can follow various inheritance patterns, including autosomal dominant, autosomal recessive, X-linked dominant, or maternal mitochondrial. The onset of symptoms may occur anywhere from childhood to late adulthood [78]. Pathogenic mutations in SPGs primarily lead to the progressive degeneration of upper motor neurons (UMNs), including the corticospinal tracts, resulting in leg spasticity, weakness, hypertonia, and hyperreflexia. These forms of HSP are referred to as Uncomplicated or Pure HSP. However, neurodegeneration can affect only UMNs, consequently disrupting their connections with lower motor neurons (LMNs), or both UMNs and LMNs, leading to muscle atrophy and fasciculations. The involvement of LMN is typical of complicated HSP. Complicated HSP is characterized by the impairments present in uncomplicated HSP, along with additional system involvement or other neurological findings, such as ataxia, seizures, intellectual disability, dementia, muscle atrophy, extrapyramidal disturbances, or peripheral neuropathy. This dual degeneration is reminiscent of amyotrophic lateral sclerosis (ALS), with the notable exception of the preservation of sacral neurons (Onuf’s nucleus), as well as normal bladder and rectal sphincter function until the final stages of ALS [79].

Hereditary spastic paraplegia (HSP) can be inherited through all possible modes of transmission (autosomal dominant, autosomal recessive, X-linked, and maternal), with some genes showing multiple inheritance patterns. Dominantly inherited HSPs account for about 70% of all HSP cases and often manifest in adulthood [80]. The most common types are SPG4, which accounts for 40% of all autosomal dominant (AD) HSP; SPG3A, caused by a pathogenic variant in *ATL1*, which is the second most common type of AD HSP (accounting for 10–15% of all AD HSP) and is the leading cause of early-onset autosomal dominant HSP (occurring in >75% of individuals in this category); SPG30, caused by a pathogenic variant in *KIF1A*; and SPG31, caused by a pathogenic variant in *REEP1* [81,82,83,84].

Autosomal recessive (AR) HSP accounts for 25–30% of all HSP cases, with clinical symptoms often appearing in childhood [85]. The most common types of AR HSP are SPG5A, caused by pathogenic variants in *CYP7B1*, which accounts for 7.3% of all AR HSP; SPG7, caused by pathogenic variants in *SPG7* gene of chromosome 16q24.3, accounting for approximately 5% of all AR HSP; and SPG11, caused by pathogenic variants in *SPG11/KIAA1840* gene on chromosome 15p (3–5% of all AR HSP). X-linked and mitochondrial forms of HSP are the rarest, together accounting for fewer than 1–2% of all individuals affected by HSP [79,86].

## 9. MNDs with Prominent Mixed Upper and Lower Motor Neuron Involvement

### Amyotrophic Lateral Sclerosis (ALS) Associated with Presentation in Childhood

Amyotrophic lateral sclerosis (ALS) encompasses a spectrum of motor system degeneration that affects the corticospinal and corticobulbar pathways, along with motor neurons associated with cranial nerves and the anterior horn cells of the spinal cord [87]. While ALS can manifest as early as childhood, most patients experience symptoms after the age of 40. The degeneration of both upper and lower motor neurons leads to clinical features such as spasticity and muscle atrophy.

There are six recognized ALS syndromes that manifest in the first two decades of life.

ALS2 (Juvenile amyotrophic lateral sclerosis-2): This form is an autosomal recessive disorder caused by homozygous mutations in the gene encoding *alsin* on chromosome 2q33. Alsin plays a role in endosomal trafficking as a guanine nucleotide exchange factor for RAB5. Symptoms typically begin in the first decade of life, with spasticity in the face and limbs, and may include pseudobulbar effects. Progression is slow, and walking difficulties usually occur after the age of 40 [88,89].

ALS4 (Amyotrophic lateral sclerosis 4): This juvenile-onset form is an autosomal dominant distal motor neuronopathy caused by heterozygous mutations in the *senataxin* (*SETX*) gene on chromosome 9q34. The mutation affects DNA and RNA regulation, leading to neurodegeneration. Symptoms, such as difficulty walking, typically begin in the second decade of life (average onset at age 17). Both upper and lower motor neurons are impacted, but bulbar symptoms are rare. The disease progresses slowly, with most patients needing wheelchairs by their fifth or sixth decade, although they usually maintain a normal life expectancy [90,91].

ALS5: Accounting for 40% of autosomal recessive juvenile ALS cases, ALS5 is caused by homozygous or compound heterozygous mutations in the *spatacsin* gene (*SPG11*) on chromosome 15q21. Onset typically occurs between ages 7 and 23, with a mean onset age of 16. Patients present with limb spasticity, facial and bulbar involvement, amyotrophy, and weakness [92,93].

ALS6 (*FUS* gene mutation): This form is caused by mutations in the *FUS* gene on chromosome 16p11 and is characterized by rapid progression of spastic gait, dysarthria, and lower motor neuron dysfunction. It is inherited in either an autosomal dominant or recessive manner [26,94].

ALS6-21 (Juvenile recessive ALS): An autosomal recessive form associated with mutations on chromosomes 6p25 and 21q22, it is identified in a single consanguineous family. Symptoms begin between ages 4 and 10 and feature upper motor neuron involvement, distal muscle wasting in the limbs, and bulbar dysfunction [95,96].

ALS16 (Juvenile amyotrophic lateral sclerosis-16): This form is caused by homozygous mutations in the *SIGMAR1* gene on chromosome 9p13. ALS16 is characterized by slowly progressive lower limb spasticity and weakness, with symptoms typically appearing between ages 1 and 2 [95,97].

Currently, there is no curative treatment for juvenile amyotrophic lateral sclerosis. Despite this, advances in clinical management—particularly through the establishment of multidisciplinary specialized clinics—have significantly improved both survival rates and quality of life for adult ALS patients [98]. These clinics focus on comprehensive supportive care, addressing various aspects of the disease, such as respiratory support, nutrition, mobility aids, and psychological assistance, all of which are essential in managing the symptoms and slowing the progression of ALS. Although this supportive approach has not been fully optimized for juvenile cases, it represents a key model that could potentially be adapted for younger patients to improve outcomes [99].

One of the pivotal therapeutic insights in adult ALS involves cortical hyperexcitability, a pathophysiological hallmark that contributes to motor neuron degeneration. This hyperexcitability is partly mitigated by riluzole, the only approved disease-modifying therapy for ALS, which has shown modest success in prolonging survival and slowing disease progression [100,101]. However, this aspect of ALS pathophysiology—cortical hyperexcitability—has not yet been thoroughly investigated in juvenile ALS. Given that ALS may have distinct biological characteristics, understanding whether similar mechanisms are at play could be critical for developing targeted therapies.

The landscape of ALS research is evolving, with promising developments in understanding the molecular and genetic underpinnings of the disease. Gene-based therapies are increasingly emerging as potential treatment strategies. In April 2023, a novel antisense oligonucleotide (ASO)-based therapy called tofersen received accelerated approval from the U.S. Food and Drug Administration (FDA) for treating adults with amyotrophic lateral sclerosis (ALS) linked to mutations in the *SOD1* gene which accounts for about 20% of familial ALS cases and up to 2% of sporadic cases (Figure 2) [102]. As an ASO, tofersen consists of short, synthetic RNA or DNA strands that bind to a complementary sequence, modifying mRNA expression. Specifically, tofersen targets and degrades *SOD1* mRNA produced by mutated SOD1 genes, thereby reducing the production of the harmful SOD1 protein. This mutated protein is toxic to motor neurons and astrocytes, which play key roles in the onset and progression of ALS. By lowering SOD1 protein levels in cerebrospinal fluid (CSF) and blood, tofersen helps slow disease progression and improves the quality of life for patients with *SOD1*-related ALS [103,104].

These ASO therapies are now progressing through early-phase clinical trials for certain ALS genetic subtypes, offering hope that targeted molecular interventions could soon be available [105].

## 10. Conclusions

Motor neuron diseases display a broad clinical and genetic spectrum, posing diagnostic challenges.

Research into motor neuron disorders has advanced significantly through the identification of genes associated with these conditions, highlighting the genetic and phenotypic complexities of the diseases. Disruption of normal axonal function is a common feature of motor neuron diseases, and the discovery of more genes responsible for familial forms continues to support early observations of motor neuron vulnerability. While many genes have been linked to motor neuron degeneration, genetic heterogeneity reveals that the condition results from disruptions across various cellular systems, with some cases still lacking identifiable mutations in known genes.

As research progresses, the limitations of traditional diagnostic categories are becoming apparent, with some forms of motor neuron diseases, such as SMA-PLUS, demonstrating broader central nervous system involvement beyond motor neurons. The phenotypic variability seen in mutations also underscores the difficulty in categorizing these disorders and suggests that both inherited and sporadic forms share overlapping genetic factors.

The advent of next-generation sequencing (NGS) has transformed the diagnostic landscape, improving the accuracy and efficiency of identifying genetic causes of motor neuron diseases. This technology has expanded our understanding of the genetic and clinical spectrum, highlighting the importance of screening for rare genetic variants and structural abnormalities. While effective treatments remain limited, the growing knowledge of genetic underpinnings opens the door to personalized therapeutic strategies, with a focus on targeting specific mutations to slow the progression of motor neuron degeneration. As understanding deepens, interpreting genetic data becomes more feasible, offering hope for better diagnostic and therapeutic outcomes in the future.

## Figures and Tables

**Table 1:** Principal inherited pediatric motor neuron diseases with known gene variants.

	Type	Onset	Gene	Location	Inheritance
**MN diseases with prominent lower motor neuron involvement**					
Classical infantile spinal muscular atrophy	Spinal muscular atrophy (SMA 0)	Antenatal	*SMN1*	5q13	AR
	Spinal muscular atrophy (SMA 1) Werdnig–Hoffman disease	Neonatal	*SMN1*	5q13	AR
	Spinal muscular atrophy (SMA 2) Dubowitz disease	Infancy	*SMN1*	5q13	AR
	Spinal muscular atrophy (SMA 3) Kugelbert–Welander disease	Infancy	*SMN1*	5q13	AR
MNDs with prominent lower motor neuron involvement with additional features (Sma-like or Sma-plus or atypical Sma)					
Distal infantile SMA with diaphragm paralysis (DSMA1 or SMARD1)		Neonatal	*IGHMBP2*	11q13.3	AR
SMA with respiratory failure 2 (SMARD2)		Neonatal	*LAS1L*	Xq12	X-linked
SMA with arthrogryposis		Antenatal Neonatal	*SMN1*	5q13	AR
		Antenatal Neonatal	*TRIP4*	15q22.31	AR
		Antenatal Neonatal	*ASCC1*	10q22.1	AR
		Antenatal Neonatal	*UBA1*	Xp11.3	X-linked
Scapuloperoneal spinal muscular atrophy (SPSMA)		Infancy to childhood	*TRPV4*	12q23-q24.1	AD
X-linked spinal muscular atrophy-2 (SMAX2)		Birth or infancy	*UBE1*	Xp11	X-linked recessive
Lethal Arthrogryposis with Anterior Horn Cell Disease (LAAHD) or Congenital Arthrogryposis with Anterior Horn Cell Disease (CAAHD)		Antenatal	*GLE1*	9q34.11	AR
Lethal Congenital Contracture Syndrome 1 (LCCS1) or Multiple Contracture Syndrome, Finnish Type		Antenatal	*GLE1*	9q34.11	AR
Lethal Congenital Contracture Syndrome 2 (LCCS2) or Multiple Contracture Syndrome, Israeli Bedouin Type A		Antenatal	*ERBB3*	12q13.2	AR
Lethal Congenital Contracture Syndrome 3 (LCCS3) or Multiple Contracture Syndrome, Israeli Bedouin Type B		Antenatal	*PIP5K1C*	19p13.3	AR
Lethal Congenital Contractural Syndrome 4 (LCCS4)		Antenatal	*MYBPC1*	12q23.2	AR
SMA plus syndromes linked to mitochondrial disorders		Neonatal	*SCO2*	22q13.33	AR
		Neonatal	*DGUOK*	2p13.1	AR
		Infancy	*TK2*	16q21	AR
Brown–Vialetto–Van Laere (BVVL) syndrome	BVVLS1	Infancy	*SLC52A3*	20p13	AR
	BVVLS2	Infancy	*SLC52A2*	8q24	AR
		Childhood	*UBQLN1*	9q21	AR
Fazio–Londe syndrome		Infancy	*SLC52A3*	20p13	AR
Pontocerebellar Hypoplasia with Spinal Muscular Atrophy (PCH1)	PCH 1A	Infancy	*VRK1*	14q32.2	AR
	PCH 1B	Infancy	*EXOCS3*	9p13.2	AR
	PCH1C	Infancy	*EXOCS8*	13q13.3	AR
	PCH1D	Birth/infancy	*EXOSC9*	4q27	AR
	PCH1E	Neonatal	*SLC25A46*	5q22.1	AR
		Infancy	*MORC2*	22q12.2	AD—de novo
Spinal Muscular Atrophy with Progressive Myoclonic Epilepsy (SMAPME)		Childhood	*ASAH1*	8p22	AR
Spinal Muscular Atrophy with Lower Extremity Predominance (SMA-LED)	SMA-LED1	Congenital to childhood	*DYNC1H1*	14q32.31	AD
	SMA-LED2	Congenital to childhood	*BICD2*	9q22.31	AD
Scapuloperoneal Spinal Muscular Atrophy (SPSMA)		Congenital to childhood	*TRPV4*	12q24.11	AD
**MN diseases with prominent upper motor neuron involvement**					
Hereditary spastic paraplegia (HSP)	SPG5	Childhood	*CYP7B1*	8q12	AR
	SPG7	Childhood	*SPG7*	16q24.3	AR
	SPG11	Childhood	*SPG11*	15	AR
	SPG15	Childhood or early adulthood	*ZFYVE26*	14q22-q24	AR
	SPG18	Childhood	*ERLIN2*	8p11	AR
	SPG21	Childhood	*ACP33*	15q22.31	AR
	SPG26	Childhood	*B4GALNT1*	12q13.3	AR
	SPG28	Childhood	*DDHD1*	14q22.1	AR
	SPG35	Childhood	*FA2H*	16q23.1	AR
	SPG46	Childhood	*GBA2*	9p13.3	AR
	SPG47	Childhood	*AP4B1*	1p13.2	AR
	SPG50	Childhood	*AP4M1*	7q22.1	AR
	SPG51	Childhood	*AP4S1*	14q12	AR
	SPG52	Childhood	*AP4E1*	15q21.2	AR
	SPG49	Childhood	*TECPR2*	14q32.33	AR
	SPG54	Childhood	*DDHD2*	8p11.23	AR
	SPG55	Childhood	*C12orf65*	12q24.31	AR
	SPG56	Childhood	*CYP2U1*	4q25	AR
	SPG3A	Infantile to childhood	*ATL1*	14q22.1	AD
	SPG4	Infantile to adulthood	*SPAST*	2p22.3	AD
	SPG30	Juvenile to adulthood	*KIF1A*	2q37.3	AD
**MN diseases with prominent mixed upper and lower motor neuron involvement**					
Amyotrophic Lateral Sclerosis (ALS) associated with presentation in childhood	ALS2	Infancy	*Alsin*	2q33	AR
	ALS4	Childhood	*Senataxin*	9q34	AD
	ALS5	Childhood	*Spatacsin*	15q21	AR
	ALS6	Childhood	*FUS*	16p11	AD/AR
	ALS6-21	Infancy		6p25 and 21q22	AR
	ALS16	Infancy	*SIGMAR1*	9p13	AR

## Data Availability

Data will be made available upon request to the corresponding author.
